# Complementary characterization data in support of uniaxially aligned electrospun nanocomposites based on a model PVOH-epoxy system

**DOI:** 10.1016/j.dib.2016.01.046

**Published:** 2016-02-01

**Authors:** Samaneh Karimi, Mark P. Staiger, Neil Buunk, Alison Fessard, Nick Tucker

**Affiliations:** a The New Zealand Institute for Plant & Food Research, Lincoln 7608, New Zealand; b Department of Mechanical Engineering, University of Canterbury, Private Bag 4800, Christchurch 8140, New Zealand; c The MacDiarmid Institute for Advanced Materials and Nanotechnology, P.O. Box 600, Kelburn, Wellington 6140, New Zealand; d Electrospinz Limited, 44 Lee St, Blenheim 7201, New Zealand; e Department of Ergonomy, Design and Mechanical Engineering, Université de Technologie de Belfort Montbéliard, 90400 Sevenans, France; f School of Engineering, University of Lincoln, Brayford Pool, Lincoln LN6 7TS, United Kingdom

**Keywords:** Nanocomposites, Thermal properties, Mechanical properties, Electrospinning

## Abstract

This paper presents complementary data corresponding to characterization tests done for our research article entitled “Uniaxially aligned electrospun fibers for advanced nanocomposites based on a model PVOH-epoxy system” (Karimi et al., 2016) [1]. Poly(vinyl alcohol) and epoxy resin were selected as a model system and the effect of electrospun fiber loading on polymer properties was examined in conjunction with two manufacturing methods. A novel electrospinning technology for production of uniaxially aligned nanofiber arrays was used. A conventional wet lay-up fabrication method is compared against a novel, hybrid electrospinning–electrospraying approach. The structure and thermomechanical properties of resulting composite materials were examined using scanning electron microscopy, dynamic mechanical analysis, thermogravimetric analysis, differential scanning calorimetry, Fourier transform infrared spectroscopy, and tensile testing. For discussion of obtained results please refer to the research paper (Karimi et al., 2016) [1].

**Specifications Table**

**Table:** 

Subject area	Engineering, material science, nanotechnology
More specific subject area	Nanocomposite materials, electrospinning
Type of data	Table, image, graph, figure
How data was acquired	SEM (JEOL Neoscope JCM-5000, USA), tensile ((Instron 4444), DMA (TA Instruments Q800), TGA (TA Instruments Q600), DSC (TA Instruments Q2000), FTIR (Bruker ALPHA series spectrometer equipped with an ALPHA platinum ATR single-reflection diamond ATR module)
Data format	Raw, analyzed
Experimental factors	No pretreatment on samples was done
Experimental features	Tensile tests were done according to ASTM D882. Other tests were performed according to common practices and fully explained in the research paper [1].
Data source location	Lincoln, New Zealand
Data accessibility	Data found in this article

**Value of the data**

• The huge potential of electrospun sub-micron aligned fiber arrays in reinforcing a composite material is demonstrated which can open up new possibilities in various fields.
• The importance of nanocomposite manufacturing practice in final performance of the article is demonstrated.
• The data are useful for comparing purposes when addressing the influence of aligned electrospun fibers for reinforcing articles.

## 1 Data

**Fig. 1:**
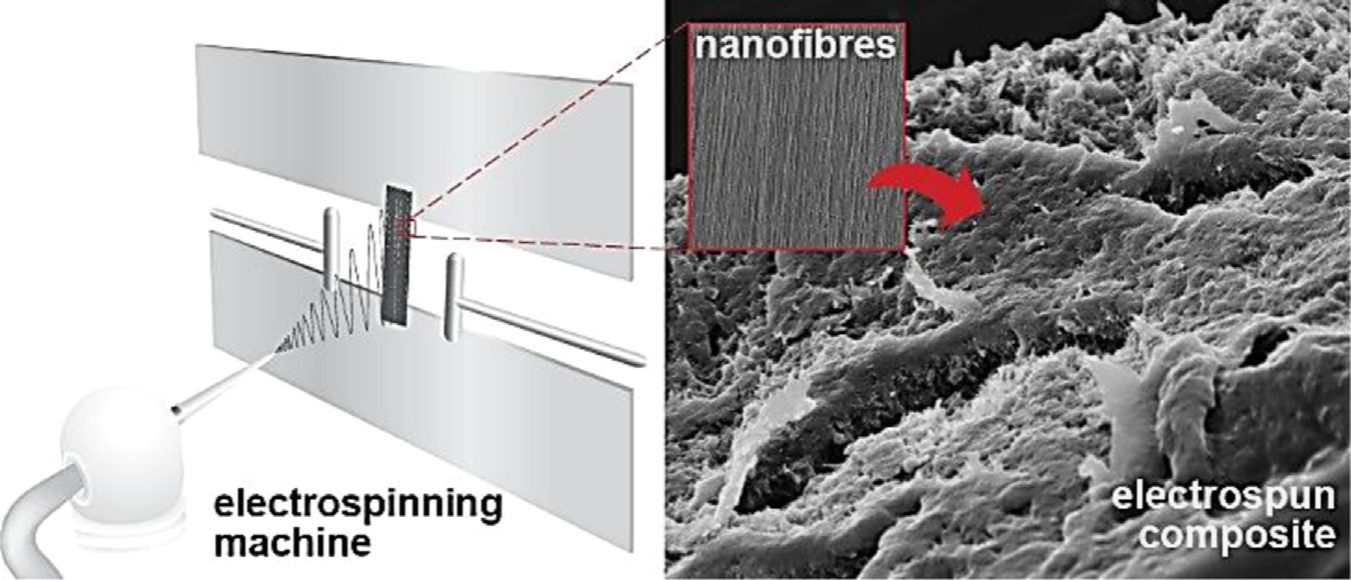
Schematic representation of nanocomposite fabrication.

A schematic representation of nanocomposite fabrication is presented in Fig. 1.

**Fig. 2:**
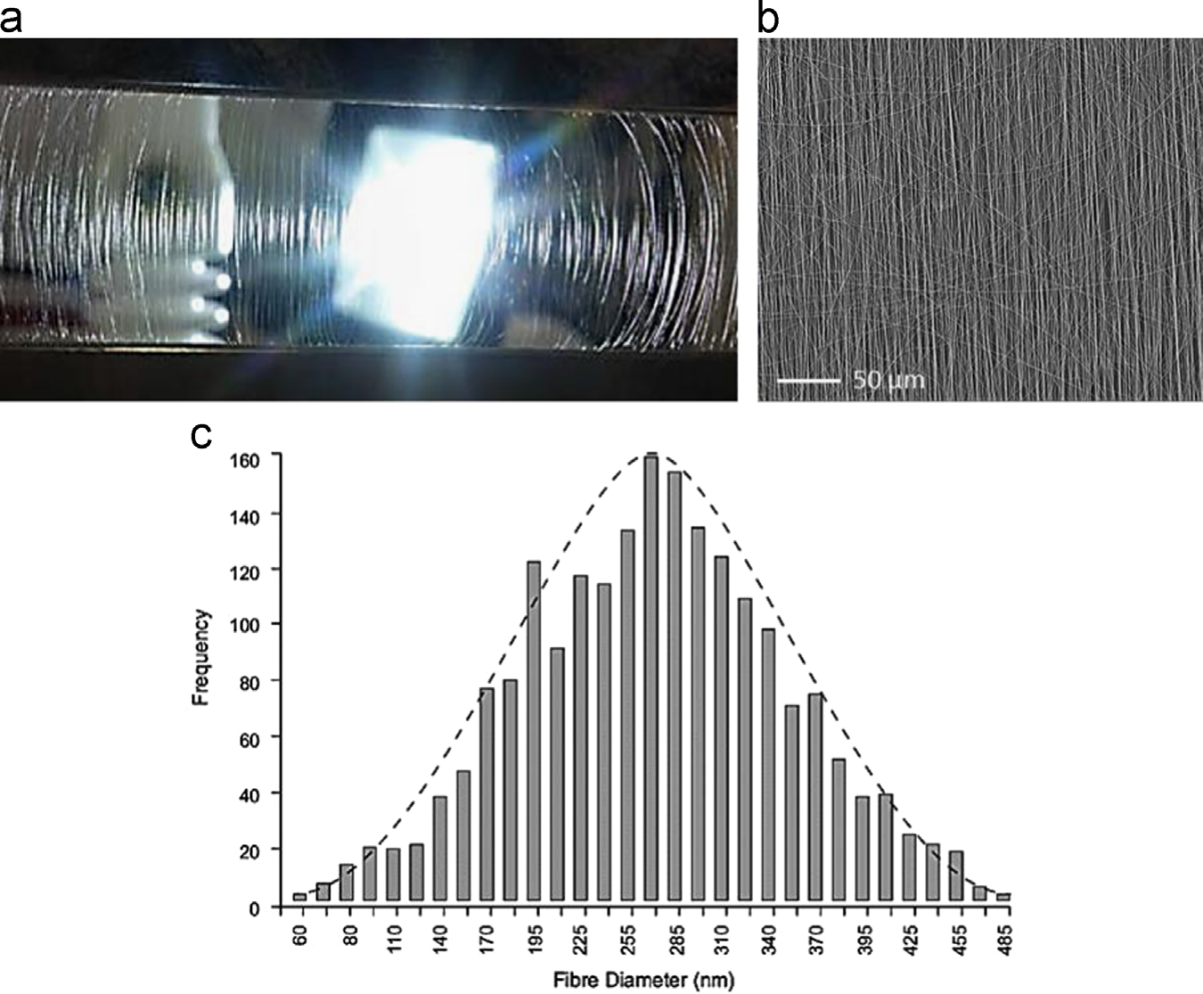
(a) Photograph of aligned fibers before collection (b) SEM of aligned fibers and (c) diameter distribution of obtained fibers.

**Fig. 3:**
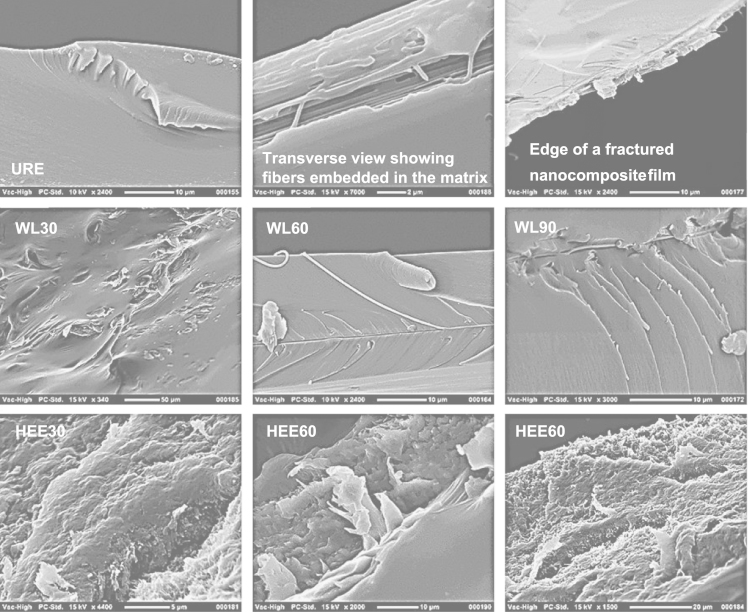
SEM micrographs of fracture surfaces of fabricated materials.

SEM micrograph and image analysis results of electrospun fibers are illustrated in Fig. 2. Original micrographs obtained from SEM of fracture surfaces of fabricated nanocomposites are presented to show the material microstructure and quality (Fig. 3).

Tensile test data obtained for all specimens and the corresponding stress–strain diagrams is depicted (Fig. 5).

Table 1 is presented a complete range of obtained tensile, DMA and TGA data.

TG and DTG curves are depicted in Fig. 6.

DSC curves are presented in Fig. 7.

FTIR graphs of all studied materials are displayed in Fig. 8.

## 2 Experimental design, materials and methods

Poly (vinyl alcohol) being chosen for the fiber, and epoxy resin for the matrix. The materials used in this study were selected based on processability and successful use in the manufacture of macro-scale composites [2]. Two different composite manufacturing methods were explored. Firstly; conventional wet lay-up followed by vacuum consolidation, and secondly deposition of the uncured epoxy matrix into the reinforcement layer-by-layer using electrospraying, followed by vacuum consolidation. For detailed information please refer to the related research paper [1].

### 2.1 Scanning electron microscopy (SEM)

Specimens were sputter-coated with gold for 240 s to avoid charging. The average diameter of the PVOH fiber was based on the measurement of ~200 fibers by computer image analysis of scanning electron micrographs (Electrospinz SEM Analyser software, Electrospinz Ltd., New Zealand) [3] as depicted in Fig. 2. The SEM of fractured surfaces of fabricated materials are presented in Fig. 3.

### 2.2 Tensile test

**Fig. 4:**
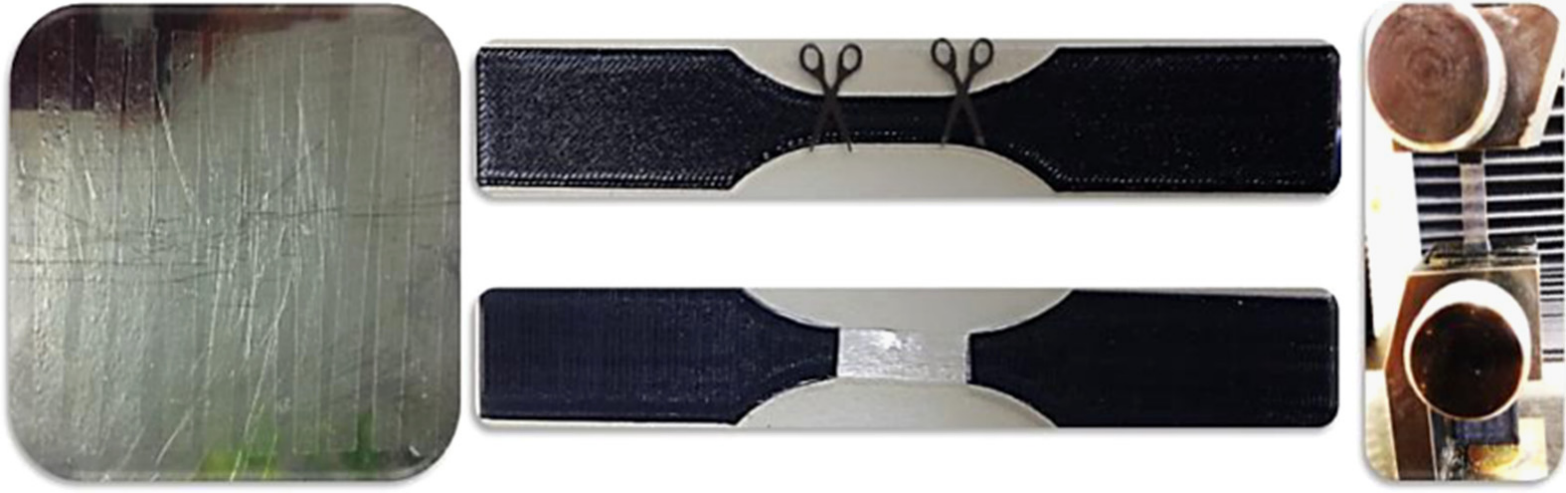
Tensile test sample preparation and clamping.

**Fig. 5:**
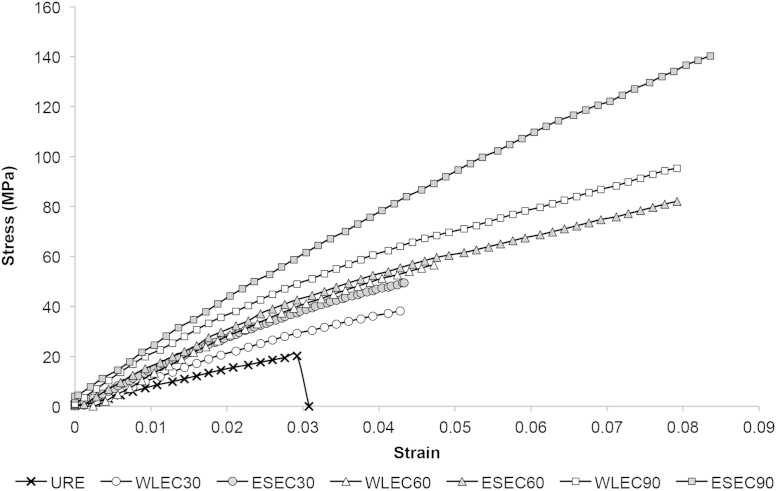
Engineering stress–strain curves of fabricated materials.

Rectangular coupons (80(*l*)×6(*w*)×0.1(*t*) mm) were tested at room temperature using a gauge length of 25 mm in accordance with ASTM D882 using a crosshead speed of 50 mm/min. Data were averaged over at least 4 replicates. A pair of 3D printed dumb bell-shaped tensile specimens were also prepared from polylactide to provide a non-slip gripping surface during the testing of the composite films (Fig. 4). The obtained engineering stress–strain curves are presented in Fig. 5.

### 2.3 Thermogravimetric analysis

**Fig. 6:**
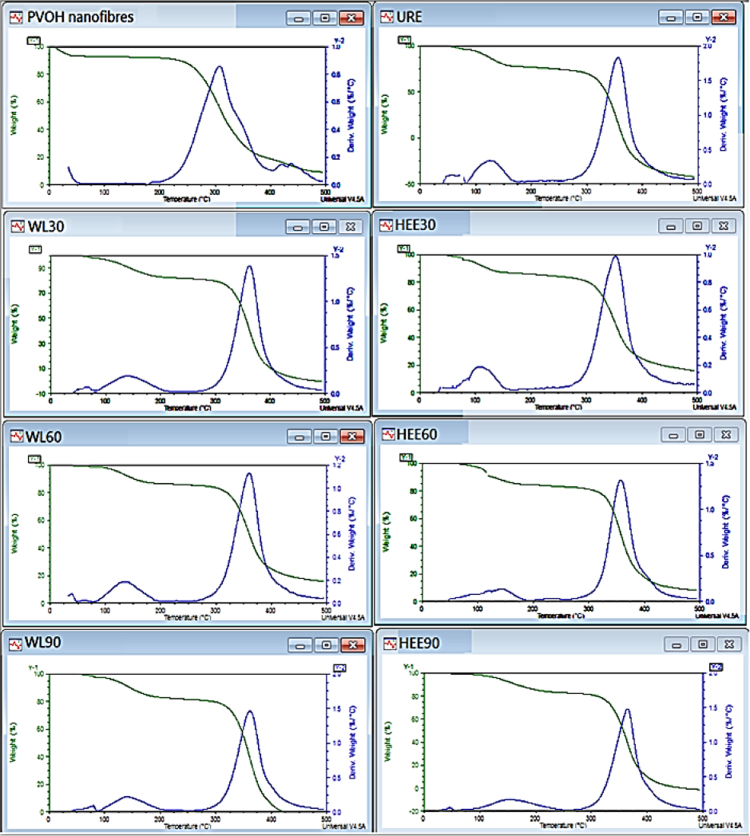
Obtained TG and DTG curves.

Specimens of ~3 mg were placed in platinum pans and heated from 20 to 500 °C at a heating rate of 10 °C/min under a nitrogen atmosphere. Obtained graphs are illustrated in Fig. 6.

### 2.4 Dynamic mechanical analysis

DMA was carried out within the linear viscoelastic range of the samples in tensile mode using rectangular specimens (15(*l*)×5(*w*)×0.1(*t*) mm). The storage (E’) and loss (E”) moduli were measured at a frequency of 1 Hz over a temperature range of 20 to 150 °C. The temperature ramp rate was 5 °C/min. A summary of obtained data pertaining DMA, TGA and tensile tests are presented in Table 1.

### 2.5 Differential scanning calorimetry

**Fig. 7:**
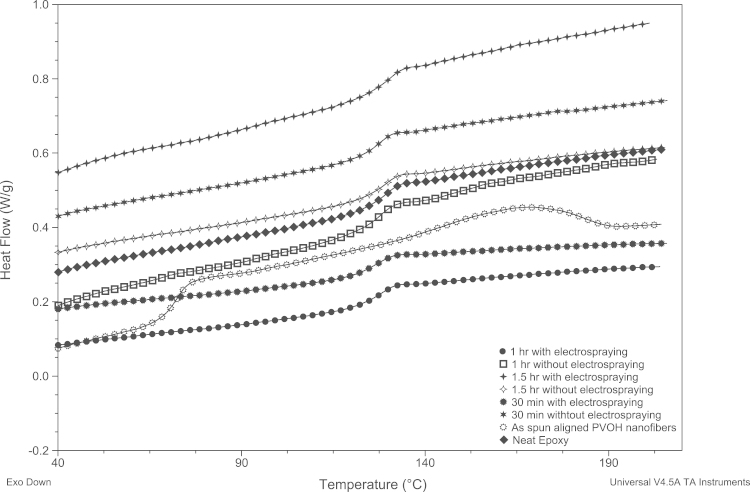
Superposition of obtained DSC graphs (second heat).

Specimens of ~1.5 mg were cut to lay flat on the base of the low mass Tzero aluminum pan. To remove prior thermal history, specimens were first heated for 10 min, then cooled, and then reheated in the temperature range of 20–250 °C at a heating rate of 10 °C/min. Superposition of obtained DSC graphs (second heat) is shown in Fig. 7.

### 2.6 Fourier transform infrared spectroscopy

**Fig. 8:**
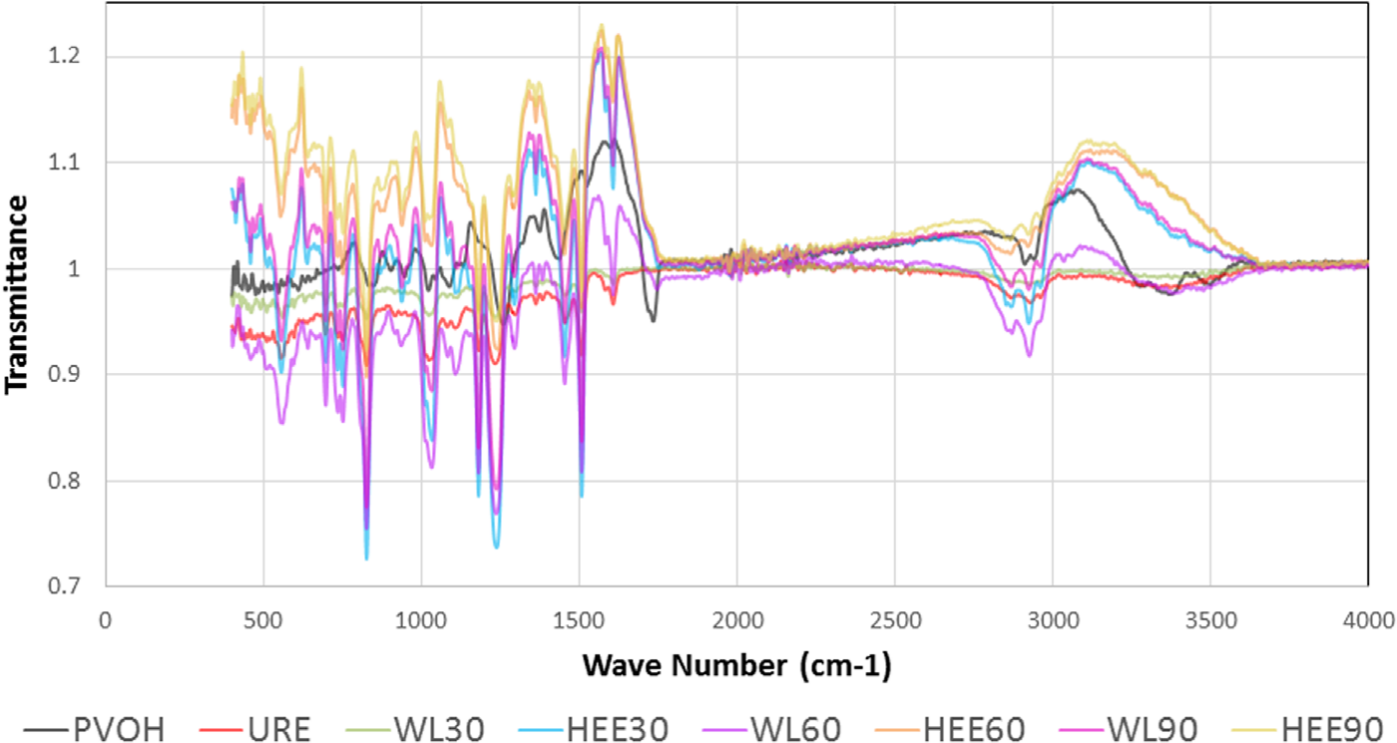
Superposition of obtained FTIR graphs.

FT-IR was carried out on the electrospun mat, unreinforced epoxy and nanocomposites using a Bruker ALPHA series spectrometer equipped with an ALPHA platinum ATR single-reflection diamond ATR module. Spectra were averaged over 16 scans using a resolution of 2 cm^−1^ over the mid-IR range (4000–400 cm^−1^). The data was analyzed using OPUS software (Bruker). Superposition of obtained FTIR graphs is presented in Fig. 8.

## Figures and Tables

**Table 1:** Summary of Tensile, DMA and TGA data.

	**Treatments**	URE	WL30	HEE30	WL60	HEE60	WL90	HEE90
**Tensile**	Tensile strength (MPa)	20.21	38.28	49.50	56.73	82.15	95.38	140.40
Young׳s modulus (GPa)	0.91	1.23	1.43	1.73	1.90	2.13	2.24
Elongation at break (%)	2.92	4.28	4.52	4.88	7.92	8.12	8.36

**DMA**	Storage modulus at 55 °C (MPa)	394	479	579	672	1068	1101	1124
Storage modulus at 75 °C (MPa)	20	32	78	190	340	565	776
tan*δ* peak temperature (°C)	68	71	73	81	83	84	92
tan*δ* peak magnitude	0.78	0.74	0.59	0.56	0.52	0.46	0.45

**TGA**	Onset of degradation (°C)	326	328	329	330	332	334	340
Temperature of maximum decomposition rate (*T*_max_) (°C)	355	357	359	358	360	362	364
